# Pediatric retroperitoneal non-organ-originated malignancies: An analysis based on SEER database

**DOI:** 10.1097/MD.0000000000034910

**Published:** 2023-10-06

**Authors:** Wei Shen, Hongqiong Geng, Yin Zhou, Xinghai Yang

**Affiliations:** a Department of Pediatric Surgery, Maternal and Child Health Hospital of Hubei Province, Tongji Medical College, Huazhong University of Science and Technology, Wuhan City, P.R. China.

**Keywords:** pediatric malignancies, prognosis, retroperitoneal, SEER, treatment

## Abstract

Retroperitoneal non-organ-originated malignancies are rare pediatric tumors with challenging diagnosis and treatment. The present study aimed to analyze the clinicopathological characteristics, treatment, and prognosis of retroperitoneal non-organ-originated malignancies. In the study, we included the pathological diagnosis of pediatric retroperitoneal non-organ-originated malignant tumors between 2000 to 2019 through the updated Surveillance, Epidemiology, and End Results database. We use the Kaplan–Meier survival curve to calculate the overall survival (OS) and cancer-specific survival (CSS). The risk of all-cause death and disease-specific death were analyzed using Cox proportional hazard regression model and Fine-and-Grey competitive hazard model, respectively. In the study, a total of 443 pediatric retroperitoneal non-organ-originated malignancies were included. Of them, only 22.3% of patients had no metastatic disease, 42.9% had distant metastasis and 34.8% had locally advanced diseases. The primary pathological tumor was neuroblastoma followed by germ cell tumor. The overall 10-year OS and CSS were 70.7% and 73.1%, respectively, and the 10-year OS and CSS of metastatic diseases were 54.4% and 56.6%, respectively. Older children, worse tumor stage at diagnosis, incomplete resection, and prolonged time from diagnosis to treatment were significantly associated with worse survival outcomes. Radiotherapy and chemotherapy did not significantly improve the prognosis of patients without complete tumor resection. The study indicated that most pediatric retroperitoneal non-organ-originated malignancies diagnosed with metastatic diseases have plagued treatment. Radiotherapy and chemotherapy are the main treatment methods for children unable to undergo complete surgical treatment. However, these treatments do not reach the same therapeutic effect as complete tumor resection after early diagnosis. Hence, early diagnosis and surgery for complete tumor resection are of utmost importance.

## 1. Introduction

Primary retroperitoneal solid tumors are rare and divided into three categories based on their origin: mesodermal, neurogenic, and extragonadal germ cell tumors.^[1]^ Generally, early retroperitoneal lesions are usually asymptomatic and difficult to be diagnosed early.^[1]^ As the clinical symptoms of retroperitoneal tumors lack specificity, imaging examination is crucial for early diagnosis.^[2]^ Computed tomography and magnetic resonance imaging can be used to clarify the boundary of the tumor and its relationship with adjacent tissues. It is an ideal method for the localization diagnosis of retroperitoneal tumors.^[3,4]^ Retroperitoneal tumors are diverse and complex pathological types,^[5,6]^ including benign tumors, borderline malignant tumors, and low-grade to high-grade malignant tumors.^[7]^ Retroperitoneal malignant tumors easily adhere to the surrounding organs and tissues; therefore, damage to important blood vessels and organs is a risk during surgery. Hence, multidisciplinary cooperation, including gastrointestinal surgery, hepatobiliary surgery, vascular surgery, urology, and orthopedics, is necessary to ensure successful operation.

Currently, studies on retroperitoneal primary tumors are focused more on adult patients^[7–11]^ than pediatric patients, resulting in insufficient evidence for the pediatric retroperitoneal non-organ-originated malignancies.^[12–14]^ Moreover, due to limited incidences and a small number of cases included in most studies, it is difficult to systematically summarize the clinicopathological features, treatment, prognosis, and prognostic analysis of pediatric patients with retroperitoneal tumors. To this end, the present study aimed to analyze the clinicopathological features, treatment, and prognosis of pediatric patients with malignant retroperitoneal tumors, and the risk factors affecting its prognosis based on the updated surveillance, epidemiology, and end results (SEER) database from 2000 to 2019.

## 2. Patients and methods

### 2.1. Data source

All data were obtained from the SEER database of 18 registries (https://seer.cancer.gov/). All procedures of this study were performed in accordance with the Declaration of Helsinki and the approval arrangement of the Ethics Committee of the Maternal and Child Health Hospital of Hubei Province, Tongji Medical College, Huazhong University of Science and Technology. Patients with primary non-organ-originated retroperitoneal malignancy of neuroblastoma and other uncommon histology were retrospectively identified from 2000 to 2019. Inclusion criteria were as follows: Patients < 19 years. All diagnoses are confirmed by histology, and not by autopsy or death certification. Patients with the complete cause of death and follow-up data. Cases with large missing information were excluded.

### 2.2. Study variables

The following primary variables were included for data analysis: year of diagnosis (2000–2004, 2005–2009, 2010–2014, and 2015–2019), age at diagnosis (0–1 year, 1–4 years, 4–11 years and 11–19 years), sex (male and female), race (white, black and others), median household income ($0–$59,999, $60,000–$69,999 and ≥$70,000), residence (metropolitan and nonmetropolitan), histology (neuroblastoma and others), tumor size (≤8 cm and ≥ 8 cm), tumor stage (distant, localized and regional), surgery and approaches (none, local tumor destruction/excision, simple/partial surgical removal of primary site and total/radical surgical removal of primary site), surgery for none primary metastatic site (yes and no), lymph node examination (none, 1–2 node, 3 + node), radiotherapy and chemotherapy (yes and no) and months to treatment (0 and ≥ 1).

### 2.3. Statistical analyses

In a normal distribution, the continuous variables were described as mean ± standard deviation, and compared using a Student *t* test. In a non-normal distribution, the data were described as median and interquartile range, and compared using Wilcoxon rank-sum test. Classification variables were represented by frequency (%) and compared using the Chi-square test. Overall survival (OS) and cancer-special survival (CSS) were the primary events of interest in this study. OS and CSS were calculated using the Kaplan–Meier survival curve. The hazard ratio (HR), sub-distribution hazard ratio (sHR), and corresponding 95% confidence interval (95% CI) of all-cause mortality and cancer-specific death were calculated using Cox proportional hazard model and the Fine–and–Gray competitive hazard model, respectively. All analyses were conducted using R 42.0, and a *P* value of < .05 (two-sided) was statistically significant.

## 3. Results

### 3.1. Baseline characteristics

A total of 443 children with retroperitoneal malignant tumors were included in this study. Of them, 42.9%, 22.3%, and 34.8% of patients had metastatic diseases, localized diseases, and regional diseases, respectively, at the time of diagnosis (Table 1). The proportion of infants < 1-year-old with metastatic diseases was lower than those with localized and regional diseases (18.4% vs 42.4% vs 31.2%). In addition, the proportion of neuroblastoma in groups of metastases and regional disease was higher than that in the localized disease group (74.7% vs 72.1% vs 56.6%). The proportion of patients with metastatic diseases receiving radiotherapy and chemotherapy increased, whereas the proportion of patients who received surgical treatment was less than that in the localized and regional diseases (Table S1, Supplemental Digital Content, http://links.lww.com/MD/J629).

**Table 1:** Baseline characteristics of study cohort by tumor stage.

	ALL	By stage				
		Localized	Regional	Distant	*P*.overall	*P*.trend
	443	99 (22.3)	154 (34.8)	190 (42.9)		
Year at diagnosis, N (%)						
2000–2004	107 (24.2)	24 (24.2)	38 (24.7)	45 (23.7)	.906	.653
2005–2009	119 (26.9)	23 (23.2)	43 (27.9)	53 (27.9)		
2010–2014	108 (24.4)	28 (28.3)	38 (24.7)	42 (22.1)		
2015–2019	109 (24.6)	24 (24.2)	35 (22.7)	50 (26.3)		
Age at diagnosis, Median (IQR)	2.00 [0.00;5.00]	1.00 [0.00;4.50]	1.00 [0.00;5.00]	3.00 [1.00;5.75]	.001	.006
Age at diagnosis, N (%)						
0–1 year	125 (28.2)	42 (42.4)	48 (31.2)	35 (18.4)	.001	.074
1–4 year	191 (43.1)	32 (32.3)	66 (42.9)	93 (48.9)		
4–11 year	69 (15.6)	13 (13.1)	18 (11.7)	38 (20.0)		
11–19 year	58 (13.1)	12 (12.1)	22 (14.3)	24 (12.6)		
Sex, N (%)						
Female	215 (48.5)	58 (58.6)	74 (48.1)	83 (43.7)	.055	.359
Male	228 (51.5)	41 (41.4)	80 (51.9)	107 (56.3)		
Race, N (%)						
White	340 (76.7)	75 (75.8)	122 (79.2)	143 (75.3)	.623	.477
Black	55 (12.4)	10 (10.1)	18 (11.7)	27 (14.2)		
Other	48 (10.8)	14 (14.1)	14 (9.09)	20 (10.5)		
Median household incomes, N (%)						
$0–$59999	126 (28.4)	30 (30.3)	44 (28.6)	52 (27.4)	.907	.526
$60000–$69999	148 (33.4)	31 (31.3)	55 (35.7)	62 (32.6)		
$70000+	169 (38.1)	38 (38.4)	55 (35.7)	76 (40.0)		
Region, N (%)						
Metropolitan	403 (91.0)	92 (92.9)	138 (89.6)	173 (91.1)	.667	.671
Nonmetropolitan	40 (9.03)	7 (7.07)	16 (10.4)	17 (8.95)		
Histology, N (%)						
Neuroblastoma	309 (69.8)	56 (56.6)	111 (72.1)	142 (74.7)	.005	.488
Other	134 (30.2)	43 (43.4)	43 (27.9)	48 (25.3)		
Tumor size (mm), Median (IQR)	88.5 [64.0;125]	85.0 [65.0;125]	82.5 [60.0;124]	100 [69.0;124]	.285	.115
Tumor size, N (%)						
≤ 8 cm	155 (35.0)	37 (37.4)	61 (39.6)	57 (30.0)	.292	.11
8 + cm	207 (46.7)	48 (48.5)	65 (42.2)	94 (49.5)		
Unknown	81 (18.3)	14 (14.1)	28 (18.2)	39 (20.5)		
Months to treatment, N (%)						
0	336 (75.8)	83 (83.8)	112 (72.7)	141 (74.2)	.103	.83
1+	107 (24.2)	16 (16.2)	42 (27.3)	49 (25.8)		
Surgical treatment, N (%)						
None	93 (21.0)	11 (11.1)	22 (14.3)	60 (31.6)	<.001	.002
Local tumor excision/destruction	95 (21.4)	37 (37.4)	30 (19.5)	28 (14.7)		
Simple/partial surgical removal of primary site	94 (21.2)	19 (19.2)	37 (24.0)	38 (20.0)		
Total/radical surgical removal of primary site	161 (36.3)	32 (32.3)	65 (42.2)	64 (33.7)		
Surgery for non-primary other distant sites, N (%)						
None	396 (89.4)	98 (99.0)	146 (94.8)	152 (80.0)	<.001	<.001
Yes	47 (10.6)	1 (1.01)	8 (5.19)	38 (20.0)		
Lymphnodes examined, N (%)						
None	252 (56.9)	71 (71.7)	73 (47.4)	108 (56.8)	.001	.654
2 nodes	74 (16.7)	14 (14.1)	32 (20.8)	28 (14.7)		
3 + node	72 (16.3)	11 (11.1)	35 (22.7)	26 (13.7)		
Unknown	45 (10.2)	3 (3.03)	14 (9.09)	28 (14.7)		
Radiotherapy, N (%)						
No	345 (77.9)	86 (86.9)	137 (89.0)	122 (64.2)	<.001	<.001
Yes	98 (22.1)	13 (13.1)	17 (11.0)	68 (35.8)		
Chemotherapy, N (%)						
No/unknown	114 (25.7)	56 (56.6)	51 (33.1)	7 (3.68)	<.001	<.001
Yes	329 (74.3)	43 (43.4)	103 (66.9)	183 (96.3)		

IQR = interquartile range.

**Figure 1.**
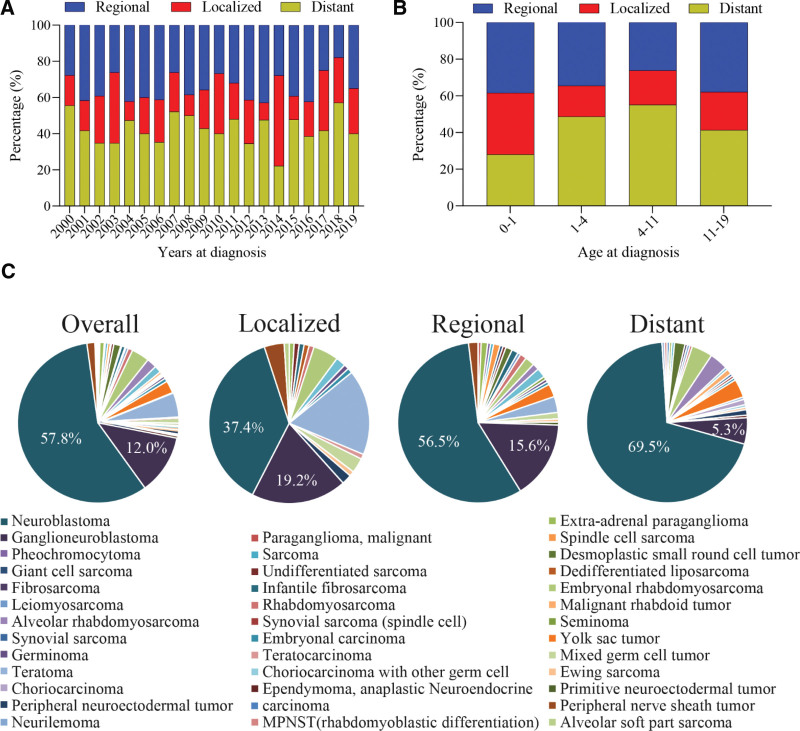
The proportions of each tumor stage and type. (A) The proportion of regional, localized, and distant diseases at different years of diagnosis. (B) The proportion of regional, localized, and distant diseases in patients of different ages at diagnosis. (C) The proportion of tumor types.

Figure 1A shows the proportion of tumor stages at diagnosis from the year 2000 to 2019. The proportion of patients with metastatic diseases had no obvious trend of increase or decrease. However, patients in the < 1 year old group were less likely to have metastatic disease at diagnosis than those in 1 to 4 years, 4 to 11 years, and 11 to 19 years groups (28.0% vs 48.7% vs 55.1% vs 41.4%; Fig. 1B). Neuroblastoma was the most common malignant tumor in pediatric retroperitoneum followed by germ cell tumors (Fig. 1C).

### 3.2. Survival outcomes

**Figure 2.**
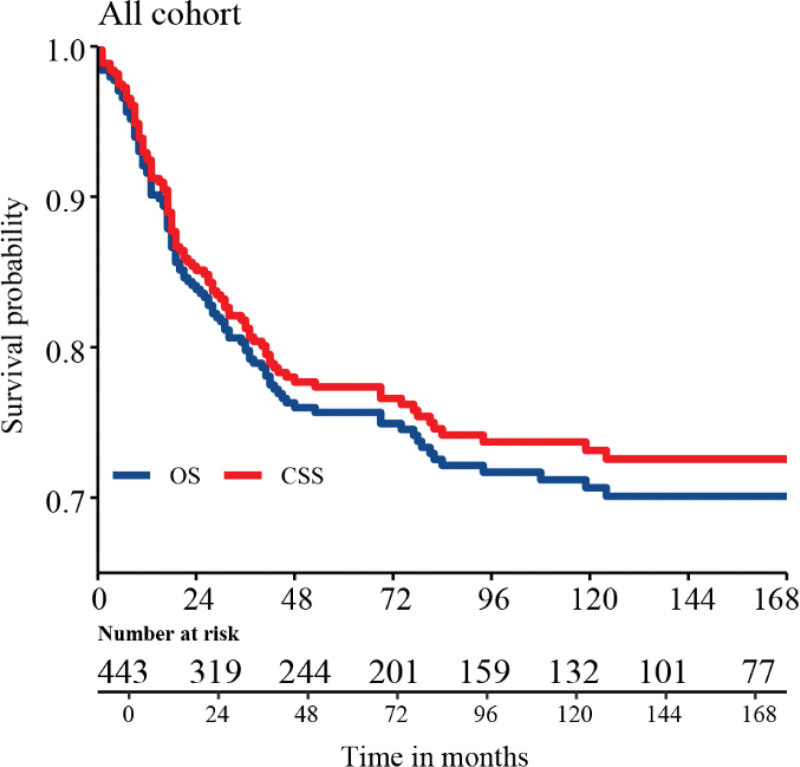
Overall survival (OS) and cancer-specific survival (CSS) curve using Kaplan–Meier survival curve analysis.

Figure 2 shows the OS and CSS of the overall population. After a median follow-up of 96 months, the overall population did not reach the median OS and CSS. Table 2 shows that the 5-year and 10-year OS were 75.7% and 70.0%, respectively. Whereas, the 5-year and 10-year CSS were 77.4% and 73.1%, respectively. The 5-year OS and CSS improved in 2010 to 2014 (OS: 78.7%; CSS: 80.5%) compared to that in 2000 to 2004 (OS: 70.1%; CSS: 72.2%). OS and CSS decreased with age at diagnosis. The 10-year OS and CSS decreased from 91.4% for infants < 1-year-old to 46.6% for adolescents aged 11 to 19 years. In addition, it was observed that the greater the tumor size, the worse the prognosis. The 10-year OS of tumors ≤ 8 cm and > 8 cm were 81.5% and 64.5%, respectively. The prognosis of metastatic diseases is often worse than that of lesions without organ metastasis, and the prognosis of patients without surgery or treatment with radiotherapy and chemotherapy alone is often unsatisfactory.

**Table 2:** Five-, and 10-years OS and CSS for different subgroups.

	5-OS [95% CI]	10-OS [95% CI]	5-CSS [95% CI]	10-CSS [95% CI]
All cohort	75.7 [71.5–80.1]	70.7 [66.0–75.7]	77.4 [73.3–81.7]	73.1 [68.6–0.78]
Year at diagnosis, N (%)				
2000–2004	70.1 [61.9–79.3]	68.2 [59.9–77.6]	72.2 [64.1–81.3]	71.2 [63.0–80.5]
2005–2009	75.2 [67.8–83.5]	68.3 [60.2–77.5]	75.2 [67.8–83.5]	68.3 [60.2–77.5]
2010–2014	78.7 [71.2–87.1]		80.5 [73.1–88.6]	
2015–2019				
Age at diagnosis				
0–1 year	91.4 [86.4–96.7]	91.4 [86.4–96.7]	92.3 [87.5–97.3]	92.3 [87.5–97.3]
1–4 year	73.4 [67.0–80.4]	68.6 [61.6–76.5]	75.9 [69.6–82.7]	70.9 [63.9–78.7]
4–11 year	69.8 [59.9–82.6]	59.6 [47.4–74.9]	71.1 [60.3–83.8]	60.7 [48.5–76.1]
11–19 year	57.9 [45.7–73.2]	46.6 [33.1–65.7]	50.0 [46.7–74.4]	54.7 [40.5–72.2]
Sex				
Female	79.0 [73.4–84.9]	75.4 [69.2–82.2]	80.2 [74.7–86.1]	78.5 [72.7–84.8]
Male	72.7 [66.7–79.2]	66.3 [59.5–73.8]	74.8 [68.9–81.2]	68.2 [61.4–75.7]
Race				
White	75.6 [70.9–80.7]	71.1 [65.9–76.7]	77.4 [72.8–82.4]	73.9 [68.8–79.3]
Black	76.1 [65.1–89.0]	72.8 [60.9–87.1]	78.4 [67.8–90.6]	75.0 [63.3–88.8]
Other	75.7 [63.5–90.4]	55.9 [34.0–92.0]	75.7 [63.5–90.4]	55.9 [34.0–92.0]
Median household incomes				
$0-$59,999	80.0 [72.8–88.0]	75.7 [67.5–84.8]	81.3 [74.2–89.1]	78.4 [70.5–87.1]
$60,000-$69,999	72.8 [65.6–80.7]	66.4 [58.2–75.7]	74.0 [66.8–81.8]	68.9 [60.9–77.9]
$70,000+	75.2 [68.5–82.5]	71.1 [63.8–79.2]	77.6 [71.1–84.7]	73.4 [66.1–81.4]
Region				
Metropolitan	75.2 [70.8–79.9]	70.1 [65.2–75.5]	77.1 [72.7–81.7]	72.9 [68.0–78.0]
Nonmetropolitan	79.7 [68.0–93.3]	75.3 [62.0–91.3]	79.7 [68.0–93.3]	75.3 [62.0–91.3]
Histology				
Neuroblastoma	77.4 [72.5–82.6]	73.3 [67.9–79.2]	79.4 [74.6–84.4]	75.8 [70.5–81.5]
Other	71.9 [64.3–80.3]	64.9 [56.2–74.9]	73.0 [65.5–81.4]	67.4 [58.9–77.1]
Tumor size				
≤8 cm	83.6 [77.6–90.2]	81.5 [74.9–88.6]	85.4 [79.5–91.8]	84.3 [78.1–91.0]
8 + cm	72.5 [66.2–79.3]	64.5 [57.2–72.9]	73.7 [67.5–80.5]	65.6 [58.2–74.0]
Unknown	68.7 [58.9–80.3]	64.5 [53.9–77.2]	71.5 [61.8–82.7]	69.7 [59.8–81.3]
Months to treatment				
0	78.8 [74.3–83.6]	73.6 [68.4–79.2]	80.5 [76.1–85.2]	76.3 [71.3–81.6]
1+	65.1 [55.9–75.9]	60.9 [50.9–72.8]	66.8 [57.6–77.5]	62.5 [52.4–74.5]
Stage				
Distant	60.0 [52.9–68.1]	54.4 [46.9–63.1]	62.4 [55.2–70.5]	56.6 [48.9–65.4]
Localized	94.2 [89.4–99.3]	89.6 [82.1–97.8]	94.2 [89.4–99.3]	89.6 [82.1–97.8]
Regional	83.3 [77.3–89.6]	78.8 [71.9–86.3]	84.7 [79.0–90.8]	82.5 [76.3–89.3]
Surgical treatment				
None	59.1 [49.5–70.7]	55.6 [45.6–67.7]	64.3 [54.5–75.8]	60.4 [50.1–72.7]
Local tumor excision/destruction	84.4 [76.8–92.6]	80.0 [70.9–90.1]	84.4 [76.8–92.6]	80.0 [70.9–90.1]
Simple/partial surgical removal of primary site	77.3 [68.6–87.3]	72.6 [62.5–84.3]	77.3 [68.6–87.3]	74.9 [65.3–85.8]
Total/radical surgical removal of primary site	79.3 [73.0–86.3]	73.3 [66.0–81.4]	80.6 [74.4–87.4]	75.6 [68.5–83.4]
Surgery for non-primary other distant sites				
None	76.8 [72.5–81.4]	72.0 [67.1–77.3]	78.7 [74.5–83.2]	74.9 [70.1–80.0]
Yes	66.2 [53.6–81.8]	59.6 [46.1–77.0]	66.2 [53.6–81.8]	59.6 [46.1–77.0]
Lymphnodes examined				
None	74.5 [68.9–80.4]	70.0 [63.8–76.7]	76.5 [71.0–82.4]	72.6 [66.5–79.2]
2 nodes	70.7 [60.3–82.9]	68.4 [57.5–81.2]	70.7 [60.3–82.9]	68.4 [57.5–81.2]
3 + node	87.2 [79.3–96.0]	81.2 [70.6–93.4]	89.0 [81.6–97.1]	86.6 [78.2–95.9]
Unknown	72.1 [59.3–87.7]	60.1 [45.2–79.9]	74.4 [61.8–89.6]	62.0 [47.0–81.9]
Radiotherapy				
No	79.0 [74.6–83.8]	76.5 [71.8–81.6]	81.3 [77.0–85.9]	79.3 [74.7–84.1]
Yes	63.7 [54.2–75.0]	48.6 [37.3–63.4]	63.7 [54.2–75.0]	51.2 [40.0–65.4]
Chemotherapy				
No/Unknown	97.0 [93.7–100.0]	93.0 [87.0–99.5]	97.0 [93.7–100.0]	94.9 [89.9–100.0]
Yes	68.5 [63.4–74.1]	63.2 [57.5–69.3]	70.7 [65.6–76.2]	65.7 [60.1–71.8]

CI = confidence interval, CSS = cancer-special survival, HR = hazard ratio, OS = overall survival, sHR = sub-distribution hazard ratio.

### 3.3. Risk factors for prognosis

Table 3 shows the risk factors for all-cause mortality and disease-specific mortality using the Cox proportional model and Fine–Gray model. The year at diagnosis, age at diagnosis, tumor stage, surgical treatment, chemotherapy, and months to treatment are independent predictors of all-cause death and disease-specific death. Patients diagnosed between 2015 to 2019 showed improved OS [HR: 0.45, 95% CI: (0.23–0.89), *P* < .001] and CSS [sHR: 1.07, 95% CI: (1.03–1.11), *P* < .001] than those diagnosed between 2000 to 2004. Older age showed worse OS [1–4 years vs 0–1 years, HR: 3.11, 95% CI: (1.59–6.07), *P* < .001; 4–11 years vs 0–1 years, HR: 3.43, 95% CI: (1.63–7.21), *P* < .001; 11–19 years vs 0–1 years, HR: 4.61, 95% CI: (2.24–9.47), *P* < .001] and CSS [1–4 years vs 0–1 years, sHR: 3.45, 95% CI: (1.67–7.11), *P* < .001; 4–11 years vs 0–1 years, sHR: 3.70, 95% CI: (1.68–8.14), *P* < .001; 11–19 years vs 0–1 years, sHR: 5.24, 95% CI: (2.39–11.49), *P* < .001)] than the new-born baby and younger age. Lower tumor stage showed improved OS [localized vs distant, HR: 0.27, 95% CI: (0.12–0.63), *P* = .002; regional vs distant, HR: 0.58, 95% CI: (0.37–0.91), *P* = .018] and CSS [localized vs distant, sHR: 0.22, 95% CI: (0.10–0.48), *P* < .001; regional vs distant, sHR: 0.42, 95% CI: (0.27–0.67), *P* < .001]. Further, patients treated with surgery, particularly those who received total/radical surgical removal of primary site, showed improved OS [total/radical surgical removal of primary site vs none, HR: 0.60, 95% CI: (0.37–0.96), *P* = .033] and CSS [total/radical surgical removal of primary site vs none, sHR: 0.53, 95% CI: (0.31–0.90), *P* = .019] than those who received no surgical treatment. Late treatment showed worse OS [1 + months vs 0 months, HR: 1.65, 95% CI: (1.02–2.38), *P* = .041] and CSS [1 + vs 0, sHR: 1.61, 95% CI: (1.07–2.56), *P* = .025] than early treatment. Patients who did not undergo surgery and whose tumors were staged with metastatic disease were often treated with chemotherapy, and those who did not undergo chemotherapy were more likely to undergo surgery for tumor resection (Table S1, Supplemental Digital Content, http://links.lww.com/MD/J629). Hence, the patients who received chemotherapy showed a worse prognosis than those who did not (Table 3).

**Table 3:** The hazard ratio, sub-distribution hazard ratio of all-cause mortality, and cancer-specific death.

	All-cause mortality				Cancer-special mortality			
	Univariable*		Multivariable*		Univariable†		Multivariable†	
	HR (95% CI)	*P*	HR (95% CI)	*P*	sHR (95% CI)	*P*	sHR (95% CI)	*P*
Year at diagnosis, N (%)								
2000–2004	1 reference		1 reference		1 reference		1 reference	
2005–2009	0.90 [0.56–1.43]	.656	0.64 [0.38–1.07]	.086	1.01 [0.63–1.64]	.961	0.75 [0.43–1.30]	.31
2010–2014	0.80 [0.48–1.34]	.394	0.67 [0.37–1.18]	.167	0.78 [0.45–1.35]	.38	0.59 [0.30–1.14]	.11
2015–2019	0.63 [0.33–1.21]	.163	0.45 [0.23–0.89]	.021	0.54 [0.26–1.11]	.096	0.36 [0.16–0.82]	.014
Age at diagnosis (Continuous variable)‡	1.08 [1.04–1.11]	<.001	1.07 [1.04–1.10]	<.001	1.08 [1.04–1.11]	<.001	1.07 [1.03–1.11]	<.001
Age at diagnosis (categorical variable)‡								
0–1 year	1 reference		1 reference		1 reference		1 reference	
1–4 year	3.57 [1.86–6.85]	<.001	3.11 [1.59–6.07]	<.001	3.87 [1.89–7.89]	<.001	3.45 [1.67–7.11]	<.001
4–11 year	3.92 [1.90–8.09]	<.001	3.43 [1.63–7.21]	<.001	4.45 [2.04–9.73]	<.001	3.70 [1.68–8.14]	<.001
11–19 year	5.97 [2.93–12.15]	<.001	4.61 [2.24–9.47]	<.001	6.28 [2.89–13.65]	<.001	5.24 [2.39–11.49]	<.001
Sex								
Female	1 reference		1 reference		1 reference		1 reference	
Male	1.34 [0.92–1.96]	.13	0.92 [0.60–1.40]	.69	1.40 [0.94–2.10]	.097	0.96 [0.60–1.53]	.86
Race								
White	1 reference		1 reference		1 reference		1 reference	
Black	0.92 [0.51–1.65]	.772	0.75 [0.40–1.40]	.368	0.95 [0.51–1.74]	.857	0.79 [0.40–1.55]	.49
Other	1.13 [0.62–2.08]	.688	1.26 [0.63–2.51]	.508	1.26 [0.69–2.33]	.452	1.40 [0.65–3.00]	.39
Median household incomes								
$0–$59,999	1 reference		1 reference		1 reference		1 reference	
$60,000–$69,999	1.35 [0.83–2.20]	.232	1.34 [0.76–2.36]	.315	1.41 [0.84–2.36]	.192	1.65 [0.94–2.93]	.084
$70,000+	1.19 [0.73–1.94]	.492	1.21 [0.67–2.17]	.528	1.18 [0.70–1.99]	.532	1.41 [0.77–2.57]	.26
Region								
Metropolitan	1 reference		1 reference		1 reference		1 reference	
Nonmetropolitan	0.95 [0.50–1.82]	.882	1.49 [0.70–3.18]	.304	1.07 [0.55–2.05]	.848	1.89 [0.84–4.24]	.12
Histology								
Neuroblastoma	1 reference		1 reference		1 reference		1 reference	
Other	1.33 [0.90–1.96]	.153	0.92 [0.57–1.48]	.722	1.38 [0.92–2.07]	.121	1.00 [0.60–1.64]	.99
Tumor size								
≤8 cm	1 reference		1 reference		1 reference		1 reference	
8 + cm	1.92 [1.21–3.05]	.006	1.48 [0.89–2.47]	.13	2.24 [1.36–3.71]	.002	1.91 [1.10–3.31]	.022
Unknown	2.01 [1.16–3.49]	.012	1.23 [0.67–2.25]	.501	2.19 [1.20–3.98]	.01	1.60 [0.84–3.06]	.15
Months to treatment								
0	1 reference		1 reference		1 reference		1 reference	
1+	1.75 [1.17–2.62]	.006	1.65 [1.02–2.38]	.041	1.77 [1.16–2.71]	.008	1.61 [1.07–2.56]	.025
Stage								
Distant	1 reference		1 reference		1 reference		1 reference	
Localized	0.15 [0.07–0.34]	<.001	0.27 [0.12–0.63]	.002	0.17 [0.08–0.37]	<.001	0.22 [0.10–0.48]	<.001
Regional	0.41 [0.27–0.63]	<.001	0.58 [0.37–0.91]	.018	0.39 [0.24–0.61]	<.001	0.42 [0.27–0.67]	<.001
Surgical treatment								
None	1 reference		1 reference		1 reference		1 reference	
Local tumor excision/destruction	0.34 [0.19–0.62]	<.001	0.63 [0.34–1.18]	.15	0.41 [0.22–0.75]	.004	0.74 [0.38–1.45]	.381
Simple/partial surgical removal of primary site	0.49 [0.29–0.85]	.011	0.55 [0.32–0.96]	.034	0.55 [0.31–0.98]	.042	0.59 [0.32–1.09]	.9
Total/radical surgical removal of primary site	0.48 [0.30–0.75]	<.001	0.60 [0.37–0.96]	.033	0.51 [0.31–0.83]	.007	0.53 [0.31–0.90]	.019
Surgery for non–primary other distant sites								
None	1 reference		1 reference		1 reference		1 reference	
Yes	1.65 [0.98–2.77]	.058	0.88 [0.49–1.59]	.674	1.85 [1.10–3.12]	.021	1.05 [0.56–1.97]	.87
Lymphnodes examined								
None	1 reference		1 reference		1 reference		1 reference	
2 nodes	1.07 [0.65–1.77]	.781	1.24 [0.71–2.16]	.444	1.20 [0.72–2.00]	.477	1.45 [0.84–2.49]	.18
3 + node	0.55 [0.29–1.04]	.067	0.87 [0.43–1.77]	.7	0.50 [0.25–1.02]	.056	0.87 [0.40–1.89]	.73
Unknown	1.39 [0.79–2.43]	.255	1.21 [0.65–2.26]	.552	1.34 [0.73–2.45]	.338	1.15 [0.56–2.35]	.7
Radiotherapy								
No	1 reference		1 reference		1 reference		1 reference	
Yes	2.01 [1.36–2.99]	<.001	1.31 [0.78–2.19]	.307	2.24 [1.49–3.37]	<.001	1.31 [0.76–2.26]	.34
Chemotherapy								
No/Unknown	1 reference		1 reference		1 reference		1 reference	
Yes	5.97 [2.78–12.84]	<.001	2.95 [1.29–6.74]	.01	7.62 [3.10–18.73]	<.001	3.64 [1.33–9.97]	.012

CI = confidence interval, HR = hazard ratio, Shr = Sub-distribution hazard ratio.

* Cox proportional risk regression model.

† Fine and Grey regression model.

‡ Separately into the multivariate regression model.

## 4. Discussion

The present study showed that approximately 70% of the patients with pediatric primary retroperitoneal malignant tumors had neurogenic tumors, with more than half of the patients diagnosed with neuroblastoma. Most patients with retroperitoneal neuroblastoma have metastatic diseases at the time of diagnosis. In addition, neuroblastoma accounts for a higher proportion of metastatic diseases than other histology types, leading to substantial difficulty in subsequent surgical treatment. The proportion of patients with metastatic diseases at the time of consultation fluctuated at approximately 40% over the past 20 years with moderate change. The proportion of children > 1 year old with metastatic diseases at the time of diagnosis was 40%. This incidence is higher than that of children diagnosed within 1 year of age. However, the patients diagnosed between 2015 and 2019 showed a reduced risk of death compared with those diagnosed between 2000 and 2004 after adjusting for other confounding factors, thus indicating an improvement in treatment strategies. The onset of pediatric retroperitoneal tumors mainly occurs before the age of 4 years, particularly in children < 1 year of age. Multivariate regression analysis adjusted for confounding factors showed that the age of children was an independent predictor of prognosis. The risk of death increased by approximately 7% for every one-year increase in the age of patients. The risk of all-cause mortality and tumor-specific mortality was higher in children > 1 year compared with those < 1 year.

Pediatric primary retroperitoneal neuroblastoma was the primary type of tumor identified in this study. It is an embryonic tumor of the sympathetic nervous system produced by sympathetic nerve cells from the neural crest.^[15]^ It is one of the most common solid tumors in children and the most common malignant tumor in infants. Varieties of genetic characteristics in neuroblastoma, including amplification of the *MYCN* gene, a gain of *17q* chromosome, and loss of *1p* and *11q* chromosomes, affect the prognosis of the disease.^[16–19]^ Amplification of the *MYCN* gene is the most crucial prognostic factor in neuroblastoma, promoting cell proliferation, and inhibiting cell differentiation and apoptosis. It often indicates rapid disease progression in neuroblastoma, resulting in a poor prognosis. Therefore, it can be an independent prognostic indicator of neuroblastoma.^[20]^ In addition, patients with neuroblastoma can detect the expression levels of neuron-specific enolase (NSE), urinary vanillylmandelic acid (VMA), and lactate dehydrogenase (LDH), aiding in prognostic judgment.^[21–23]^ NSE is present in neurons and cells of neural origin, and is highly sensitive and specific for neuroblastoma. Elevated levels of NSE often indicate late-stage and poor prognosis. The positive rate of VMA in the urine of children with neuroblastoma and distant metastasis is higher than that of those with neuroblastoma alone. In addition, an increase in urinary VMA often indicates a poor prognosis. LDH is a crucial enzyme of the glycolytic pathway, and tumor tissues have a high metabolism. Serum LDH levels can be an important indicator of systemic tumor cell burden. In addition, an increase in LDH levels indicates a poor prognosis.

Germ cell-derived tumors were another major type of tumor identified in this study. These include malignant teratoma, seminoma, yolk sac tumor, and choriocarcinoma. Generally, extra-gonadal germ cell tumors are rare, accounting for approximately 2% of germ cell tumors.^[24]^ The most common site of extragenital germ cell tumors is mediastinum, while the second most common site is retroperitoneum.^[7]^ Evaluation of the treatment outcome is difficult due to the low incidence and limitations of the current treatment information of retroperitoneal germ cell tumors to case reports.

Although many primary retroperitoneal tumors exist and their treatments are determined accordingly, complete surgical resection is the key to ensuring a good prognosis.^[7,25]^ Since tumors originate anywhere in the vast retroperitoneal space, a standard surgical approach is non-existent to provide a reference. In clinical practice, retroperitoneal malignant tumors are often large in size with multiple distribution centers, rich in tumor blood circulation, broad base, or infiltrate surrounding organs or large blood vessels, thus making the complete surgical tumor resection difficult. During surgery, the tumor should be entirely exposed, and other organs invaded by the tumor should be carefully explored. Simultaneously, the affected organs should be removed to avoid residual tumor tissue. In addition, damage to the supply vessels of vital organs, such as iliac, mesenteric, and renal vessels, should be avoided. If damaged, the vessels should be repaired promptly to avoid ischemia and necrosis of the organs. However, if complete tumor resection is not achievable, then cytoreductive surgery for partial tumor resection should be performed for subsequent comprehensive treatment, thereby contributing to the treatment and survival of children. After adjusting other confounding factors, the study showed that patients who underwent local partial tumor resection did not have a statistically significant prognostic advantage over those who did not. However, patients who underwent complete tumor removal had a statistically significant prognostic advantage. Nevertheless, note that the surgeon’s experience, technology, and equipment conditions can determine the outcome of the surgical treatment.

The tumor stage at the time of consultation is an important indicator for determining prognosis. Considering that retroperitoneal tumors generally have local and distant metastases at the time of consultation, complete surgical resection for these children is often difficult and requires radiotherapy and chemotherapy. However, chemotherapy and radiotherapy may not provide the same therapeutic effects as complete surgical tumor resection after early diagnosis. So far, no studies have reported on the efficacy of preoperative adjuvant radiotherapy or chemotherapy for pediatric retroperitoneal tumors. The present study showed that children who received chemotherapy had a worse prognosis than those who did not, suggesting that the patients who did not receive chemotherapy may have undergone complete tumor resection earlier, resulting in a good prognosis even without chemotherapy. Treatment of malignant tumors should follow a comprehensive treatment model of multidisciplinary sharing. Hence, chemotherapy is undeniably crucial in primary systemic therapy, and its role in the treatment of neuroblastoma remains unquestioned. With the optimization of chemotherapy regimens and advancement in stem cell transplantation, immunotherapy and other technologies, clinical control and clearance of metastatic and minimal residual lesions will be significantly improved. In addition, the study observed that the time interval from diagnosis to treatment was associated with prognosis. The prognosis of children treated after more than one month was worse than those treated within one month of diagnosis, emphasizing the importance of timely treatment.

The present study has some limitations. The study was a retrospective study with inherent drawbacks. Some information was missing, including radiotherapy and chemotherapy regimen and course of treatment, patient complications after surgery, patient’s physical state before treatment, and lack of recorded prognostic indicators in blood and urine. Some information had missing values, such as tumor volume. Although we aimed to reduce the effect of selection bias in the case selection process, the study may have had selection bias in screening cases. Overall, the study achieved significant results through rigorous statistical analyses with a reasonable explanation.

## 5. Conclusion

The present study summarizes and provides a prognostic analysis of pediatric patients with retroperitoneal non-organ-originated tumors using population data. Neuroblastoma and germ cell-derived tumors were the most common types of pediatric retroperitoneal tumors. Due to the insidious onset of retroperitoneal tumors, most patients had the metastatic disease before clinical diagnosis, leading to difficulty in treatment. However, this dilemma has not changed dramatically in the past 20 years. Age at diagnosis, year of diagnosis, tumor stage, complete surgical resection, and time from diagnosis to treatment are independent risk factors that affect the prognosis of pediatric patients. Chemotherapy is the main treatment method for pediatric patients who cannot undergo complete surgery; however, it fails to achieve the same therapeutic effect as complete surgical tumor resection after early diagnosis. Hence, the aforementioned emphasizes the importance of early diagnosis and complete surgical tumor resection.

## Acknowledgments

Thank for SEER database supported by the Surveillance Research Program in the National Cancer Institute’s Division of Cancer Control and Population Sciences.

## Author contributions

**Conceptualization:** Xinghai Yang.

**Data curation:** Wei Shen.

**Formal analysis:** Wei Shen, Hongqiong Geng.

**Funding acquisition:** Xinghai Yang.

**Investigation:** Wei Shen, Xinghai Yang.

**Methodology:** Wei Shen, Yin Zhou.

**Project administration:** Xinghai Yang.

**Resources:** Xinghai Yang.

**Software:** Wei Shen, Hongqiong Geng, Yin Zhou.

**Supervision:** Xinghai Yang.

**Writing – original draft:** Wei Shen, Hongqiong Geng, Yin Zhou, Xinghai Yang.

**Writing – review & editing:** Wei Shen, Hongqiong Geng, Yin Zhou, Xinghai Yang.

## Supplementary Material
