# Supplementary material for: Pediatric retroperitoneal non-organ-originated malignancies: An analysis based on SEER database
Source: Medicine (Baltimore). 2023 Oct 6;102(40):e34910. doi: 10.1097/MD.0000000000034910 (PMC10553003; doi:10.1097/MD.0000000000034910)
Supplement: Supplementary file 1 [file medi-102-e34910-s001.docx]

| **Supplementary Table 1.** Baseline characteristics of study cohort by surgical treatment, radiotherapy, and chemotherapy. | | | | | | | | | | | | | | |
| --- | --- | --- | --- | --- | --- | --- | --- | --- | --- | --- | --- | --- | --- | --- |
|  | **By surgical treatment** | | | | |  | **By chemotherapy** | | |  | **By radiotherapy** | | | |
|  | **None** | **Local tumor excision/destruction** | **Simple/partial surgical removal of a primary site** | **Total/Radical surgical removal of a primary site** | **P** |  | **No/Unknown** | **Yes** | **P** |  | **No** | **Yes** | **P** |  |
| **N** | 93 | 95 | 94 | 161 |  |  | 114 | 329 |  |  | 345 | 98 |  |  |
| **Year at diagnosis. N (%)** |  |  |  |  | 0.005 |  |  |  | 0.595 |  |  |  | 0.1 |  |
| 2000-2004 | 27 (29.0%) | 23 (24.2%) | 17 (18.1%) | 40 (24.8%) |  |  | 26 (22.8%) | 81 (24.6%) |  |  | 90 (26.1%) | 17 (17.3%) |  |  |
| 2005-2009 | 21 (22.6%) | 17 (17.9%) | 22 (23.4%) | 59 (36.6%) |  |  | 30 (26.3%) | 89 (27.1%) |  |  | 95 (27.5%) | 24 (24.5%) |  |  |
| 2010-2014 | 20 (21.5%) | 33 (34.7%) | 23 (24.5%) | 32 (19.9%) |  |  | 33 (28.9%) | 75 (22.8%) |  |  | 76 (22.0%) | 32 (32.7%) |  |  |
| 2015-2019 | 25 (26.9%) | 22 (23.2%) | 32 (34.0%) | 30 (18.6%) |  |  | 25 (21.9%) | 84 (25.5%) |  |  | 84 (24.3%) | 25 (25.5%) |  |  |
| **Age at diagnosis. Median (IQR)** | 2.00 [0.00;7.00] | 1.00 [0.00;4.00] | 2.00 [1.00;6.00] | 2.00 [1.00;5.00] | 0.156 |  | 1.00 [0.00;4.00] | 2.00 [1.00;5.00] | 0.005 |  | 1.00 [0.00;4.00] | 4.00 [2.25;7.00] | <0.001 |  |
| **Age at diagnosis. N (%)** |  |  |  |  | 0.272 |  |  |  | 0.012 |  |  |  | <0.001 |  |
| 0-1 year | 31 (33.3%) | 32 (33.7%) | 22 (23.4%) | 40 (24.8%) |  |  | 45 (39.5%) | 80 (24.3%) |  |  | 124 (35.9%) | 1 (1.02%) |  |  |
| 1-4 year | 31 (33.3%) | 41 (43.2%) | 41 (43.6%) | 78 (48.4%) |  |  | 41 (36.0%) | 150 (45.6%) |  |  | 137 (39.7%) | 54 (55.1%) |  |  |
| 4-11 year | 14 (15.1%) | 13 (13.7%) | 19 (20.2%) | 23 (14.3%) |  |  | 18 (15.8%) | 51 (15.5%) |  |  | 47 (13.6%) | 22 (22.4%) |  |  |
| 11-19 year | 17 (18.3%) | 9 (9.47%) | 12 (12.8%) | 20 (12.4%) |  |  | 10 (8.77%) | 48 (14.6%) |  |  | 37 (10.7%) | 21 (21.4%) |  |  |
| **Sex. N (%)** |  |  |  |  | 0.219 |  |  |  | 0.119 |  |  |  | 0.352 |  |
| Female | 42 (45.2%) | 55 (57.9%) | 45 (47.9%) | 73 (45.3%) |  |  | 63 (55.3%) | 152 (46.2%) |  |  | 172 (49.9%) | 43 (43.9%) |  |  |
| Male | 51 (54.8%) | 40 (42.1%) | 49 (52.1%) | 88 (54.7%) |  |  | 51 (44.7%) | 177 (53.8%) |  |  | 173 (50.1%) | 55 (56.1%) |  |  |
| **Race. N (%)** |  |  |  |  | 0.467 |  |  |  | 0.759 |  |  |  | 0.236 |  |
| White | 74 (79.6%) | 69 (72.6%) | 74 (78.7%) | 123 (76.4%) |  |  | 90 (78.9%) | 250 (76.0%) |  |  | 271 (78.6%) | 69 (70.4%) |  |  |
| Black | 12 (12.9%) | 17 (17.9%) | 9 (9.57%) | 17 (10.6%) |  |  | 12 (10.5%) | 43 (13.1%) |  |  | 40 (11.6%) | 15 (15.3%) |  |  |
| Other | 7 (7.53%) | 9 (9.47%) | 11 (11.7%) | 21 (13.0%) |  |  | 12 (10.5%) | 36 (10.9%) |  |  | 34 (9.86%) | 14 (14.3%) |  |  |
| **Median household incomes. N (%)** |  |  |  |  | 0.013 |  |  |  | 0.361 |  |  |  | 0.552 |  |
| $0-$59999 | 21 (22.6%) | 39 (41.1%) | 24 (25.5%) | 42 (26.1%) |  |  | 36 (31.6%) | 90 (27.4%) |  |  | 100 (29.0%) | 26 (26.5%) |  |  |
| $60000-$69999 | 41 (44.1%) | 19 (20.0%) | 33 (35.1%) | 55 (34.2%) |  |  | 32 (28.1%) | 116 (35.3%) |  |  | 118 (34.2%) | 30 (30.6%) |  |  |
| $70000+ | 31 (33.3%) | 37 (38.9%) | 37 (39.4%) | 64 (39.8%) |  |  | 46 (40.4%) | 123 (37.4%) |  |  | 127 (36.8%) | 42 (42.9%) |  |  |
| **Region. N (%)** |  |  |  |  | 0.024 |  |  |  | 1 |  |  |  | 0.795 |  |
| Metropolitan | 89 (95.7%) | 81 (85.3%) | 82 (87.2%) | 151 (93.8%) |  |  | 104 (91.2%) | 299 (90.9%) |  |  | 315 (91.3%) | 88 (89.8%) |  |  |
| Nonmetropolitan | 4 (4.30%) | 14 (14.7%) | 12 (12.8%) | 10 (6.21%) |  |  | 10 (8.77%) | 30 (9.12%) |  |  | 30 (8.70%) | 10 (10.2%) |  |  |
| **Histology. N (%)** |  |  |  |  | 0.216 |  |  |  | 0.997 |  |  |  | 0.144 |  |
| Neuroblastoma | 57 (61.3%) | 71 (74.7%) | 67 (71.3%) | 114 (70.8%) |  |  | 79 (69.3%) | 230 (69.9%) |  |  | 247 (71.6%) | 62 (63.3%) |  |  |
| Other | 36 (38.7%) | 24 (25.3%) | 27 (28.7%) | 47 (29.2%) |  |  | 35 (30.7%) | 99 (30.1%) |  |  | 98 (28.4%) | 36 (36.7%) |  |  |
| **Tumor size (mm). Median (IQR)** | 89.0 [70.0;125] | 80.0 [50.0;110] | 87.5 [63.0;128] | 100 [65.0;129] | 0.028 |  | 80.0 [50.0;110] | 93.0 [68.0;126] | 0.004 |  | 83.0 [60.0;120] | 110 [80.0;138] | <0.001 |  |
| **Tumor size. N (%)** |  |  |  |  | 0.006 |  |  |  | 0.04 |  |  |  | 0.001 |  |
| ≤8 cm | 27 (29.0%) | 40 (42.1%) | 38 (40.4%) | 50 (31.1%) |  |  | 51 (44.7%) | 104 (31.6%) |  |  | 132 (38.3%) | 23 (23.5%) |  |  |
| 8+ cm | 38 (40.9%) | 37 (38.9%) | 46 (48.9%) | 86 (53.4%) |  |  | 46 (40.4%) | 161 (48.9%) |  |  | 145 (42.0%) | 62 (63.3%) |  |  |
| Unknown | 28 (30.1%) | 18 (18.9%) | 10 (10.6%) | 25 (15.5%) |  |  | 17 (14.9%) | 64 (19.5%) |  |  | 68 (19.7%) | 13 (13.3%) |  |  |
| **Months to treatment. N (%)** |  |  |  |  | 0.146 |  |  |  | 0.201 |  |  |  | 0.196 |  |
| 0 | 66 (71.0%) | 78 (82.1%) | 66 (70.2%) | 126 (78.3%) |  |  | 92 (80.7%) | 244 (74.2%) |  |  | 267 (77.4%) | 69 (70.4%) |  |  |
| 1+ | 27 (29.0%) | 17 (17.9%) | 28 (29.8%) | 35 (21.7%) |  |  | 22 (19.3%) | 85 (25.8%) |  |  | 78 (22.6%) | 29 (29.6%) |  |  |
| **Stage. N (%)** |  |  |  |  | <0.001 |  |  |  | <0.001 |  |  |  | <0.001 |  |
| Distant | 60 (64.5%) | 28 (29.5%) | 38 (40.4%) | 64 (39.8%) |  |  | 7 (6.14%) | 183 (55.6%) |  |  | 122 (35.4%) | 68 (69.4%) |  |  |
| Localized | 11 (11.8%) | 37 (38.9%) | 19 (20.2%) | 32 (19.9%) |  |  | 56 (49.1%) | 43 (13.1%) |  |  | 86 (24.9%) | 13 (13.3%) |  |  |
| Regional | 22 (23.7%) | 30 (31.6%) | 37 (39.4%) | 65 (40.4%) |  |  | 51 (44.7%) | 103 (31.3%) |  |  | 137 (39.7%) | 17 (17.3%) |  |  |
| **Surgical treatment. N (%)** |  |  |  |  |  |  |  |  | <0.001 |  |  |  | <0.001 |  |
| None |  |  |  |  |  |  | 2 (1.75%) | 91 (27.7%) |  |  | 90 (26.1%) | 3 (3.06%) |  |  |
| Local tumor excision/destruction |  |  |  |  |  |  | 38 (33.3%) | 57 (17.3%) |  |  | 75 (21.7%) | 20 (20.4%) |  |  |
| Simple/partial surgical removal of a primary site |  |  |  |  |  |  | 23 (20.2%) | 71 (21.6%) |  |  | 65 (18.8%) | 29 (29.6%) |  |  |
| Total/Radical surgical removal of a primary site |  |  |  |  |  |  | 51 (44.7%) | 110 (33.4%) |  |  | 115 (33.3%) | 46 (46.9%) |  |  |
| **Surgery for non-primary other distant sites. N (%)** |  |  |  |  | 0.116 |  |  |  | 0.001 |  |  |  | 0.001 |  |
| None | 78 (83.9%) | 89 (93.7%) | 82 (87.2%) | 147 (91.3%) |  |  | 112 (98.2%) | 284 (86.3%) |  |  | 318 (92.2%) | 78 (79.6%) |  |  |
| Yes | 15 (16.1%) | 6 (6.32%) | 12 (12.8%) | 14 (8.70%) |  |  | 2 (1.75%) | 45 (13.7%) |  |  | 27 (7.83%) | 20 (20.4%) |  |  |
| **Lymphnodes examined. N (%)** |  |  |  |  | <0.001 |  |  |  | <0.001 |  |  |  | 0.651 |  |
| None | 71 (76.3%) | 56 (58.9%) | 46 (48.9%) | 79 (49.1%) |  |  | 60 (52.6%) | 192 (58.4%) |  |  | 193 (55.9%) | 59 (60.2%) |  |  |
| 2 node | 7 (7.53%) | 14 (14.7%) | 29 (30.9%) | 24 (14.9%) |  |  | 16 (14.0%) | 58 (17.6%) |  |  | 56 (16.2%) | 18 (18.4%) |  |  |
| 3+ node | 2 (2.15%) | 22 (23.2%) | 11 (11.7%) | 37 (23.0%) |  |  | 32 (28.1%) | 40 (12.2%) |  |  | 59 (17.1%) | 13 (13.3%) |  |  |
| Unknown | 13 (14.0%) | 3 (3.16%) | 8 (8.51%) | 21 (13.0%) |  |  | 6 (5.26%) | 39 (11.9%) |  |  | 37 (10.7%) | 8 (8.16%) |  |  |
| **Radiotherapy. N (%)** |  |  |  |  | <0.001 |  |  |  | <0.001 |  |  |  |  |  |
| No | 90 (96.8%) | 75 (78.9%) | 65 (69.1%) | 115 (71.4%) |  |  | 111 (97.4%) | 234 (71.1%) |  |  |  |  |  |  |
| Yes | 3 (3.23%) | 20 (21.1%) | 29 (30.9%) | 46 (28.6%) |  |  | 3 (2.63%) | 95 (28.9%) |  |  |  |  |  |  |
| **Chemotherapy. N (%)** |  |  |  |  | <0.001 |  |  |  |  |  |  |  | <0.001 |  |
| No/Unknown | 2 (2.15%) | 38 (40.0%) | 23 (24.5%) | 51 (31.7%) |  |  |  |  |  |  | 111 (32.2%) | 3 (3.06%) |  |  |
| Yes | 91 (97.8%) | 57 (60.0%) | 71 (75.5%) | 110 (68.3%) |  |  |  |  |  |  | 234 (67.8%) | 95 (96.9%) |  |  |
| IQR: Interquartile range | | | | | | | | | | | | | | |
